# Gamified Dual-Task Training for Individuals with Parkinson Disease: An Exploratory Study on Feasibility, Safety, and Efficacy

**DOI:** 10.3390/ijerph182312384

**Published:** 2021-11-25

**Authors:** Lee-Kuen Chua, Yu-Chen Chung, David Bellard, Laura Swan, Nicole Gobreial, Amanda Romano, Ryan Glatt, Michael A. Bonaguidi, Darrin J. Lee, Yi Jin, Charles Y. Liu, Beth E. Fisher

**Affiliations:** 1 USC Neurorestoration Center, Keck School of Medicine, University of Southern California, Los Angeles, CA 90033, USA; amanda.romano@med.usc.edu (A.R.); mbonagui@usc.edu (M.A.B.); darrin.lee@med.usc.edu (D.J.L.); cliu@usc.edu (C.Y.L.); 2 Department of Physical Medicine and Rehabilitation, UT Southwestern Medical Center, Dallas, TX 75390, USA; yu-chen.chung@utsouthwestern.edu; 3 Division of Biokinesiology and Physical Therapy, University of Southern California, Los Angeles, CA 90033, USA; david.bellard@med.usc.edu (D.B.); laura.swan@med.usc.edu (L.S.); gobreial@usc.edu (N.G.); bfisher@pt.usc.edu (B.E.F.); 4 Pacific Brain Health Center, Pacific Neuroscience Institute, Santa Monica, CA 90404, USA; ryan.glatt2@providence.org; 5 Department of Stem Cell Biology and Regenerative Medicine, Eli and Edythe Broad Center for Regenerative Medicine and Stem Cell Research, Keck School of Medicine, University of Southern California, Los Angeles, CA 90033, USA; 6 Department of Biomedical Engineering, Viterbi School of Engineering, University of Southern California, Los Angeles, CA 90089, USA; 7 Department of Gerontology, University of Southern California, Los Angeles, CA 90089, USA; 8 Zilkha Neurogenetic Institute, Keck School of Medicine, University of Southern California, Los Angeles, CA 90033, USA; 9 Department of Neurological Surgery, Keck School of Medicine, University of Southern California, Los Angeles, CA 90033, USA; 10 Brain Health Leadership Foundation, Reno, NV 89509, USA; jinyi@twc.com; 11 Rancho Los Amigos National Rehabilitation Center, Downey, CA 90242, USA

**Keywords:** Parkinson’s disease, integrated dual-task training, motor-cognitive training, gamified rehabilitation, neurotechnology, physical therapy modalities, optimized intervention, exergaming, neurological rehabilitation, patient-focused intervention

## Abstract

Objectives: The feasibility and safety of the use of neurorehabilitation technology (SMARTfit^®^ Trainer system) by physical therapists in implementing a gamified physical-cognitive dual-task training (DTT) paradigm for individuals with Parkinson disease (IWPD) was examined. Additionally, the efficacy of this gamified DTT was compared to physical single-task training (STT), both of which were optimized using physio-motivational factors, on changes in motor and cognitive outcomes, and self-assessed disability in activities of daily living. Methods: Using a cross-over study design, eight participants with mild-to-moderate idiopathic PD (including one with mild cognitive impairment) completed both training conditions (i.e., gamified DTT and STT). For each training condition, the participants attended 2–3 sessions per week over 8.8 weeks on average, with the total amount of training being equivalent to 24 1 h sessions. A washout period averaging 11.5 weeks was inserted between training conditions. STT consisted of task-oriented training involving the practice of functional tasks, whereas for gamified DTT, the same task-oriented training was implemented simultaneously with varied cognitive games using an interactive training system (SMARTfit^®^). Both training conditions were optimized through continual adaptation to ensure the use of challenging tasks and to provide autonomy support. Training hours, heart rate, and adverse events were measured to assess the feasibility and safety of the gamified DTT protocol. Motor and cognitive function as well as perceived disability were assessed before and after each training condition. Results: Gamified DTT was feasible and safe for this cohort. Across participants, significant improvements were achieved in more outcome measures after gamified DTT than they were after STT. Individually, participants with specific demographic and clinical characteristics responded differently to the two training conditions. Conclusion: Physical therapists’ utilization of technology with versatile hardware configurations and customizable software application selections was feasible and safe for implementing a tailor-made intervention and for adapting it in real-time to meet the individualized, evolving training needs of IWPD. Specifically in comparison to optimized STT, there was a preliminary signal of efficacy for gamified DTT in improving motor and cognitive function as well as perceived disability in IWPD.

## 1. Background and Purpose

Parkinson disease (PD) is the second most common chronic neurodegenerative disease. It is a gradually progressive disease that affects close to one million people living in the United States and more than 10 million people globally, according to statistics published by the Parkinson’s Foundation [1]. This same source estimated that 60,000 new cases of PD are diagnosed annually in the United States alone. For individuals with Parkinson disease (IWPD), in addition to the cardinal motor symptoms (i.e., bradykinesia, rigidity, and resting tremor) that arise from dopaminergic depletion in the substantia nigra pars compacta [2], non-motor features such as cognitive deficits, psychiatric disturbance, and autonomic dysfunction are well recognized symptoms. Some of these non-motor disorders have been known to even predate the appearance of motor signs by more than a decade before a PD diagnosis can be established clinically [3]. Collectively, these motor and non-motor symptoms contribute to declined function and quality of life (QoL) [4].

Progressive cognitive impairment is one of the most common non-motor symptoms and has the most significant clinical and economic burden for the PD population [5,6]. With disease progression, Lewy pathology can become widespread, which is associated with cognitive impairment, leading to the possibility that as many as approximately 80% of IWPD in the advanced disease stage developing dementia [7,8]. Approximately one in four IWPD without dementia has mild cognitive impairment (MCI)—the earliest stage of cognitive dysfunction [9,10]. Cognitive impairment is known to affect an array of executive and non-executive (e.g., attention, information processing speed, visuospatial ability, working memory) functions in PD [11,12]. Thus, dual-task performance (essential for activities of daily living (ADL) such as walking while holding a conversation) is especially problematic when the neural correlates that are implicated in central executive function and attentional capacity are affected by PD, leading to cognitive impairment and reduced motor automaticity [13].

There is currently no cure for PD, and existing therapies such as the administration of dopaminergic medications (e.g., levodopa, bromocriptine) and deep brain stimulation can only manage disease symptomology by providing some relief for the motor manifestations of the disease. However, side effects such as dyskinesia, pulmonary valve fibrosis, and depression have been associated with the use of these therapeutics, and the efficacies of the treatments have been shown to diminish over time [14]. Even though an effective pharmacological treatment for cognitive impairment in IWPD has yet to be available [15], evidence-based, albeit limited, therapeutic benefits of challenging the impaired cognitive systems have been found [16]. As such, dual-task training (DTT) composed of physical and cognitive training elements can play an important role in the neurorehabilitation of IWPD in terms of improving their motor and cognitive function as well as their overall QoL [17,18]. In the majority of previous studies, DTT protocols were designed as the simultaneous use of a single physical task with a single verbal cognitive task [19]. A DTT program, composed of a compendium of functional and cognitive tasks targeting a wide range of motor and cognitive functions while continually being adapted to individual training needs over the entire intervention duration, has not yet been studied.

More recently, game-based interventions have been gaining increasing attention in their use of motivation and engagement to enhance rehabilitation outcomes in clinical populations. These benefits of gamification applications are primarily realized through the facilitation of rewarding experiences via the deployment of multisensory stimulation, interactivity, and fun gameplay during the rehabilitation process [20]. Consequently, the therapeutic gains of rehabilitation can be potentiated by promoting greater investment of effort into training participation, especially training involving functional tasks that are highly repetitive in nature and that can easily become boring and discouraging for patients. Patients who experience enhanced motivation and who are highly engaged during training are also more likely to adhere to the training protocol and stay committed to the training program until completion, thereby reducing dropout rates [21]. Specific to the PD population, the use of game technology developed for recreational purposes (e.g., Microsoft Kinect, Nintendo Wii) in implementing exergaming paradigms has mostly resulted in improvements in motor and cognitive outcomes, and were no worse than conventional or traditional rehabilitation [22]. Gamified technology has also been specifically developed for training purposes that aim to target specific motor and/or cognitive functional domains. However, to date, its integrated use as a rehabilitation tool in delivering DTT in PD neurorehabilitation is largely absent or limited in its technical adaptability for accommodating the different training needs of individual recipients in real-time [23]. The SMARTfit^®^ line of products is one such gamified training platform developed for multifunctional motor and cognitive training while also incorporating interaction technology with versatile configuration, but its utility for rehabilitation has yet to be tested in any clinical population (www.smartfit.rocks accessed on 1 October 2021).

The current study objectives are two-fold. Primarily, the efficacy of physical-cognitive DTT was examined by comparing its effects to those of physical single-task training (STT) on changes in motor and cognitive outcomes. Due to a reported association between perceived disability and QoL, we assessed perceived disability (measured by Movement Disorder Society-Unified Parkinson’s Disease Rating Scale Motor Aspects of Experiences of Daily Living [MDS-UPDRS II]) as a proxy measurement of QoL [24]. The second objective was to study the feasibility and safety of physical therapists’ implementation of an adaptive, gamified DTT paradigm using the SMARTfit^®^ Trainer system. To the best of our knowledge, this is the first study to utilize technology in an adaptive manner for the integration of motor and cognitive interactions to address the personalized training needs of IWPD.

## 2. Method

### 2.1. Participants

Participants with PD were recruited from a convenience sample using an online platform (Fox Trial Finder: https://www.michaeljfox.org/trial-finder accessed on 1 June 2018) during the period from 20 June 2018 to 19 July 2019. The inclusion criteria were (1) age between 50 and 85 years, (2) diagnosis of idiopathic PD using the criteria of the United Kingdom Brain Bank as determined by a movement disorders neurologist [25], (3) no contraindication to exercise, (4) being medically stable and optimized on PD medications, (5) being able to stand and ambulate independently with or without an assistive device, (6) no other neurologic, neuromuscular, orthopedic, and cardiovascular disease, and (7) medical clearance from a primary care physician to participate in this physical therapy intervention. Each IWPD provided written informed consent approved by the Institutional Review Board of the University of Southern California before participation in this study (ClinicalTrials.gov trial number: NCT03921359). All participants also signed forms to grant permission for releasing information, and the information presented herein meets the HIPAA requirements for disclosure of protected health information. A total of nine participants were recruited.

During the first visit, the participants’ demographic and clinical data were obtained (see Table 1). At the time of study enrollment, six of the nine participants were older adults (above 65 years of age); three of them had a disease duration longer than two years; two of them had moderate disease severity (Hoehn and Yahr Stage 3); one of them had MCI (for scoring below 26 points on the Montreal Cognitive Assessment) [26], mild depression (for scoring above 10 points on the Geriatric Depression Scale [Long Form]) [27], and low mobility confidence (for scoring below 80.9% on the Activities-Specific Balance Confidence scale) [28]; three of them had experienced at least one fall incident in the past six months before study participation (henceforth, described as having a faller status); and three of them were physically inactive (with less than 150 min of participation in exercise and non-exercise physical activities per day [as measured by the Longitudinal Aging Study Amsterdam Physical Activity Questionnaire], the amount that is normally attained by healthy controls) [29].

### 2.2. Procedures

The order of the two training conditions (i.e., DTT and STT) was counterbalanced across the participants. Based on a simple randomization method whereby consecutive participants were alternately assigned to one of the two orders, five participants received the DTT-STT order while four were given the STT-DTT order (see Table 1 for sequence details). For each training condition, the participants completed two to three training sessions per week over an average of 8.8 weeks. For each training condition, each participant received a summed amount of training hours that is equivalent to 24 one-hour sessions. To minimize carryover effects from the first training condition, the participants underwent a washout period averaging 11.5 weeks before starting the second training condition. A physical therapist provided one-to-one instructional supervision during every session of the two training conditions. The STT condition consisted of task-oriented trainings involving the practice of six real-life functional tasks (such as sit-to-stand and gait; see Table A1 with table format and vision parameters modified from King and Horak [2009]) [30]. For each functional task, three levels were set in five parameters (amplitude, endurance, balance, vision, and accuracy), allowing the task difficulty to be adjusted and to progress over time. Each task was practiced with quality of movement as an essential element. In contrast, the DTT condition integrated the abovementioned functional tasks with a variety of gamified cognitive tasks themed on executive function, attention, information processing speed, working memory, visuospatial ability, and language processing (see Table A2). During the DTT sessions, the SMARTfit^®^ Single (www.smartfit.rocks/products/smartfit-single accessed on 1 October 2021) and SMARTfit^®^ Strike Pods (www.smartfit.rocks/products/smartfit-strike-targets accessed on 1 October 2021) were used in tandem to facilitate visuo-tactile interaction between participants and equipment during task execution. The cognitive tasks that were performed simultaneously with these functional tasks were selectable by the physical therapist from an inventory of 16 items available in the SMARTfit^®^ Single system application. The games were customizable in terms of level of task difficulty (e.g., temporal demands, presence or absence of distractor stimuli, required level of tactile force output), duration of play, appearance (e.g., types of icon or symbol, display colors), and availability of auditory feedback. These customizable features allowed the adjustment of the degree of challenge and provided choice for supporting the autonomy of the participants. Due to the nature of the intervention involving the use of the SMARTfit® equipment in the DTT condition and not in the STT condition, the physical therapists and investigators were not blinded to the training conditions.

Of note, all of the sessions throughout the course of each training condition were optimized in several ways. These sessions were designed to be task-oriented [31] as well as tailored in real time to meet the specific training needs in terms of maintaining a challenging level according to (1) the individual capacity of at least a moderate exercise intensity of 50–70% of the individual’s maximum heart rate [32,33] and (2) individual constraints of impairments, activity limitations, and participation restrictions (if any). These training requirements were assessed by one of four physical therapists (i.e., YC, DB, LS, and NG) who were assigned on a rotational basis to supervise each session. The participants were also allowed to exercise autonomy in choosing the order that they would like to receive the three pairs of functional tasks (see Table A3 for detailed descriptions of these six tasks and their difficulty levels) [34]. Specifically, at the beginning of each round of three consecutive sessions they were asked to select one of the three pairs of functional tasks (e.g., Pair 1) to use for the current session as well as to decide on the order for the remaining two pairs of functional tasks to be used for the next two sessions (e.g., Pair 3 followed by Pair 2). In addition, the cognitive tasks to be used in the DTT condition were selected by the physical therapist with the consensus of the participants being sought in real-time. That is, when a participant preferred one cognitive task (e.g., arithmetic multiplication) to another one (e.g., arithmetic subtraction) that had been suggested by the physical therapist, the choice of the participant would be used. At each participant’s discretion, they could also rest at any time (unless otherwise stipulated for the endurance parameter of two functional tasks: multi-plane locomotion and gait) and for any duration during each session.

In order to assess the feasibility of using the SMARTfit^®^ Trainer system (SMARTfit, Inc., Camarillo, CA, USA) for delivering a gamified DTT program to participants in a challenging manner, the training duration and average heart rate (HR) data were collected for each session. Participants were required to wear an optical heart rate sensor on their forearm throughout the duration of each training session (Polar OH1, Polar Electro, Inc., New York, NY, USA). The DTT program feasibility criterion was determined by a minimum total duration of 24 h of attendance and a minimum session-average exercise intensity at the moderate level across participants. Adverse events, if any, were also monitored and recorded throughout the course of the study. Three outcome domains (i.e., motor function, cognitive function, and perceived disability) were measured before (i.e., pre-test at T0 and T2) and after (i.e., post-test at T1 and T3) each training condition using the following five instruments: (1) the Movement Disorder Society-Unified Parkinson’s Disease Rating Scale Motor Examination (MDS-UPDRS III), (2) the Modified Physical Performance Test (M-PPT), (3) the Parkinson’s Disease-Cognitive Rating Scale (PD-CRS), (4) the Trail Making Test (TMT), and (5) the MDS-UPDRS II. The participants were instructed to maintain their dopaminergic medication regimen as directed by their neurologists, and all training and assessment sessions were conducted during the “ON” state (within a period of four hours after medication).

### 2.3. Statistical Analysis

To compare the treatment responses between DTT and STT as well as changes during the washout period (i.e., differences between the end of the first training condition and the start of the second training condition), each change (or absolute) score was individually normalized to remove between-participant variability from the data [35]. Due to a low sample size that is not able to provide sufficient statistical power, the 84% confidence interval (CI) of the normalized mean change (or absolute) score of one training condition (or score of one timepoint) was used for statistical comparison with that of the other training condition (or score of another time point). In this approach, the absence of an overlap between the two 84% CIs was used as the main criterion to determine any significant differences in the training effects between DTT and STT as well as in comparing scores during the washout period (between the post-test of the first training condition and the pre-test of the second training condition). This use of the 84% CI over the 95% CI was adopted to determine statistical significance using an α value of 0.05 [36].

## 3. Results

Eight (P1–8) out of nine participants completed both training conditions. One participant (P9) completed the STT as the first training condition, but could not start the second training condition (DTT) due to the early termination of the study necessitated by the COVID-19 pandemic. The data of P9 were subsequently omitted from further analyses.

**Figure 1:**
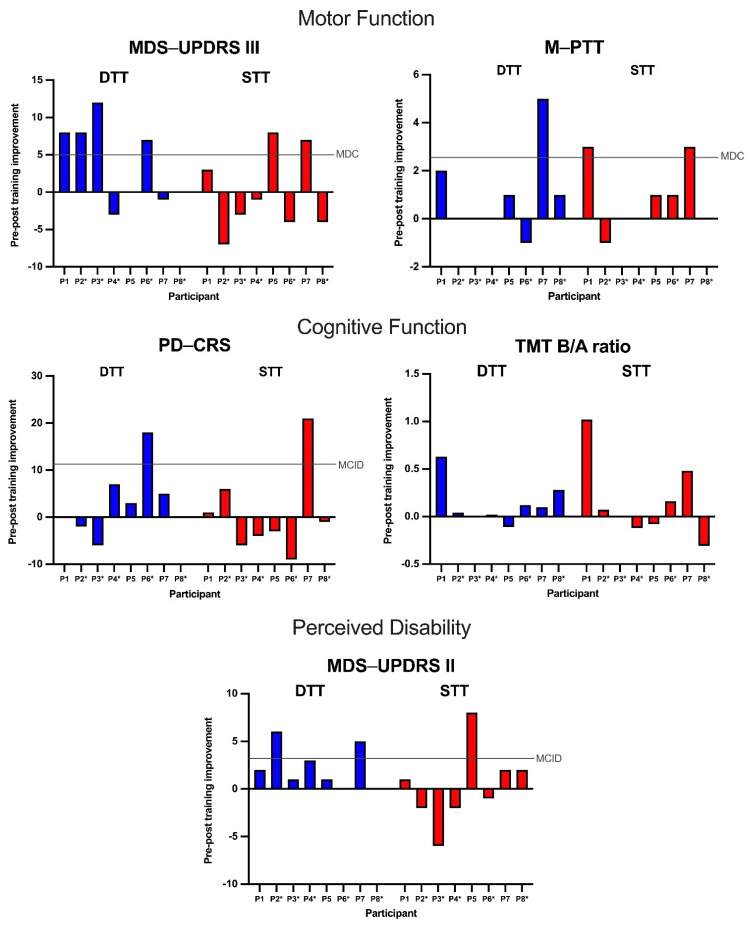
Comparison of individual raw change scores between DTT and STT for five outcome measures. Reference lines indicate values of minimal detectable change (MDC) or minimum clinically important difference (MCID); no MDC and MCID values of TMT B/A ratio have been established for adults with PD. * Participant was assigned the dual-task training condition followed by the single-task training condition.

**Figure 2:**
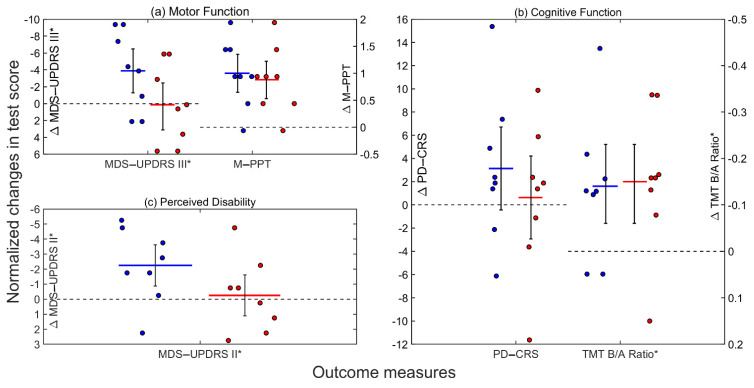
Comparison of individual normalized change scores (colored circles) and group means of normalized change scores (colored horizontal bars) between DTT and STT for five outcome measures. Whisker ends represent upper and lower bounds of 84% CIs for group means. Dashed horizontal lines represent no change in pre-post training scores; values above them indicate pre-post training improvements. * Negative values indicate score improvements for this outcome measure.

The raw and normalized change scores (from pre-test to post-test) for all of the outcome measures are shown for the eight participants in Figure 1 and Figure 2, respectively.

### 3.1. Feasibility and Safety

Each of the eight participants who were included in the analyses attended a minimum total of 24 h of training for each of the two training conditions (DTT condition averaging 18.5 ± 3.8 sessions across all participants and STT condition averaging 19.1 ± 4.3 sessions across all participants). On average, a moderate exercise intensity was achieved during the DTT sessions (averaging 61.4 ± 9.2% of HR_max_ across all participants) and the STT sessions (averaging 64.5 ± 11.8% of HR_max_ across all participants). There was no occurrence of adverse events during the study.

### 3.2. Motor Measures

#### 3.2.1. MDS-UPDRS III Score

The MDS-UPDRS is a PD severity and progress assessment (Goetz et al., 2008). The third section of the MDS-UPDRS is the motor examination subscale that includes 14 items to evaluate tremor, rigidity, bradykinesia, gait, and postural instability [37].

Four out of the eight participants improved in their MDS-UPDRS III scores after completing the DTT, with improvement exceeding the minimum detectable change (MDC) of 5 points in all four participants [38], whereas three participants showed an improvement in MDS-UPDRS III scores upon the completion of the STT, with two of them showing improvements that were above the MDC (DTT_MDC_:STT_MDC_ = 4:2). Shulman et al. (2010) further defined three categories of clinically important difference (CID) values for the MDS-UPDRS III score [39]. With reference to their minimum CID (MCID) value of 2.5 points, the same four participants surpassed the MCID after the DTT. After the STT, the same two participants who exceeded the MDC and one additional participant, making a total of three, exceeded the MCID (DTT_MCID_:STT_MCID_ = 4:3). In terms of a moderate CID value of 5.2 points, the results for the DTT and the STT are similar to those reported above using an MDC value of 5 points as the reference (DTT_ModerateCID_:STT_ModerateCID_ = 4:2). Only one participant had a score change above a large CID value of 10.8 points after the DTT, but none of the participants’ improvements reached this criterion after the STT (DTT_LargeCID_:STT_LargeCID_ = 1:0).

Across participants, the improvement seen in the MDS-UPDRS III scores for the DTT (*M*_normalized_ = −3.88 ± 4.69, 84%CI [−6.48, −1.27]) was not statistically different from no change in the MDS-UPDRS III scores for the STT (*M*_normalized_ = 0.13 ± 4.69, 84%CI [−2.84, 2.71]). During the washout period, the MDS-UPDRS III scores did not significantly change from the end of the first training condition (*M*_normalized_ = 26.88 ± 3.03, 84%CI [25.19, 28.56]) to the start of the second training condition (*M*_normalized_ = 28.25 ± 3.03, 84%CI [26.56, 29.94]).

#### 3.2.2. M-PPT Score

The M-PPT is a nine-item test that assesses multiple dimensions of physical function (basic and complex ADL) with different levels of difficulty. It is an objective assessment of the degree of frailty using a combination of seven items from the Physical Performance Test, the chair rise test, and the Romberg test [40].

Four participants improved in M-PPT, with one participant improving above an MDC value of 2.46 points (averaged from King et al. [2015] and Paschal et al. [2006]) at the end of the DTT [41,42]. In comparison, four participants had improved scores, with two of them being higher than the MDC after undergoing the STT (DTT_MDC_:STT_MDC_ = 1:2).

The improvement in the M-PPT scores across the participants was not different between the DTT paradigm (*M*_normalized_ = 1.00 ± 0.62, 84%CI (0.65, 1.35)) and the STT paradigm (*M*_normalized_ = 0.88 ± 0.62, 84%CI [0.53, 1.22]). There was no significant change in M-PPT scores between the end of the first training condition (*M*_normalized_ = 31.38 ± 0.32, 84%CI [31.20, 31.55]) and the start of the second training condition (*M*_normalized_ = 31.50 ± 0.32, 84%CI [31.32, 31.68]) during the washout period.

### 3.3. Cognitive Measures

#### 3.3.1. PD-CRS Score

The PD-CRS is a valid, reliable, and useful neuropsychological battery that is designed to cover the full spectrum of cognitive defects that are associated with PD. It includes 10 “subcortical-type” items (attention, working memory, the Stroop test, four verbal fluencies, immediate and delayed verbal memory, clock drawing), and two “cortical-type” items (naming, copy of a clock) [43].

Upon the completion of the DTT, there was an improvement in the PD-CRS scores in four participants, with one participant exceeding a MCID value of 11.5 points (averaged from a range of 10 to 13 points reported in de Bobadilla et al. [2013]) [44]. Three participants showed improvement in their PD-CRS scores, with one of them experiencing an improvement above the MCID criterion after completing the STT (DTT_MCID_:STT_MCID_ = 1:1).

Across participants, there was no change in the PD-CRS scores after the DTT program (*M*_normalized_ = 3.13 ± 6.43, 84%CI [−0.45, 6.70]) as well as after the STT program (*M*_normalized_ = 0.63 ± 6.43, 84%CI [−2.95, 4.20]), both of which did not differ statistically. During the washout period, there was no significant change in the PD-CRS scores from the end of the first training condition (*M*_normalized_ = 104.00 ± 3.96, 84%CI [101.80, 106.20]) to the start of the second training condition (*M*_normalized_ = 103.63 ± 3.96, 84%CI [101.42, 105.83]).

#### 3.3.2. TMT B/A Ratio

The TMT B/A ratio is a derived score that is obtained by dividing the time to complete Part B of the computerized TMT (PEBL Test Battery) by the time taken to complete Part A of the computerized TMT [45]. Neither a MDC nor a MCID has been established for adults with PD.

Seven participants showed improvement in the TMT B/A ratio after the DTT in comparison to only four participants who improved in the TMT B/A ratio after the STT.

There was no statistical difference between the improvement in the TMT B/A ratio across participants following the DTT (*M* = −0.14 ± 0.15, 84%CI [−0.22, −0.05]) compared to the STT (*M* = −0.15 ± 0.15, 84%CI [−0.24, −0.07]). The TMT B/A ratio did not significantly change from the end of the first training condition (*M*_normalized_ = 1.42 ± 0.18, 84%CI [1.31, 1.52]) to the start of the second training condition (*M*_normalized_ = 1.31 ± 0.18, 84%CI [1.21, 1.41]) during the washout period.

### 3.4. Perceived Disability Measure

#### MDS-UPDRS II Score

The second section of the MDS-UPDRS is the motor experiences of daily living subscale and includes 13 items for the self-assessment of disability pertaining to impairment and difficulties with ADL and instrumental ADL [37]. Six participants improved in their MDS-UPDRS II scores upon the completion of the DTT, with two of them exceeding the MCID of 3.05 points [46], whereas four participants showed improvements in their MDS-UPDRS II scores after completing the STT, with one of them improving above the MCID (DTT_MCID_:STT_MCID_ = 2:1). In terms of a categorical change in this self-reported evaluation of disability [24], four of six participants who were in the mild-disability category before DTT improved to the no-disability category after DTT compared to the one of three participants who experienced an improvement from the mild-disability category to the no-disability category after STT. A separate participant remained in the mild-disability category and maintained the same rating before and after DTT as the first training condition, but was categorized as having moderate disability after the washout period, and improved to the mild-disability category after STT as the second training condition. None of the participants belonged to the severe-disability category before the start of either of the two training conditions.

Across participants, the improvement that was observed in the MDS-UPDRS II scores for the DTT (*M*_normalized_ = −2.25 ± 2.46, 84%CI [−3.62, −0.88]) was not statistically different from no change in the MDS-UPDRS II scores for the STT (*M*_normalized_ = −0.25 ± 2.46, 84%CI [−1.62, 1.12]). During the washout period, the MDS-UPDRS II scores did not significantly change from the end of the first training condition (*M*_normalized_ = 4.25 ± 1.33, 84%CI [3.51, 4.99]) to the start of the second training condition (*M*_normalized_ = 4.50 ± 1.33, 84%CI [3.76, 5.24]).

## 4. Discussion

All of the participants were individually able to complete a total of 24 h for the gamified DTT despite varied demographic and clinical characteristics within the sample cohort. Across these participants, the average heart rate that was reached across all training sessions was equivalent to a moderate exercise intensity. No adverse events were reported during this study, indicating that the gamification of the optimized DTT using the SMARTfit^®^ Trainer system was feasible and safe for this cohort of eight IWPD.

Overall, the results of overlapping 84% CIs of the mean change scores across the eight participants showed that the effects of gamified DTT were comparable to STT on motor function (i.e., MDS-UPDRS III and M-PPT), cognitive function (i.e., PD-CRS and TMT B/A ratio), and perceived disability (i.e., MDS-UPDRS II) for this cohort. Although gamified DTT was found to be as efficacious as STT across all participants, actual score improvements (as indicated by 84% CIs of normalized changes in score not including the null value) were achieved in more outcome measures after gamified DTT (i.e., in MDS-UPDRS III, M-PPT, TMT B/A ratio, and MDS-UPDRS II) compared to after STT (i.e., in M-PPT and TMT B/A ratio). Broadly and across all three outcome domains (i.e., motor function, cognitive function, and perceived disability), five participants (P1, P2, P3, P5, and P7) were able to show improvements in their motor and cognitive function as well as in their perceived disability after undergoing gamified DTT compared to two participants (P1 and P7) who achieved improvement in all of these three outcome domains after receiving STT. Notably, after gamified DTT, all eight participants attained improvements in at least two outcome measures across two outcome domains, whereas after STT, two participants (P3 and P4) were not able to improve their scores in any of the five outcome measures.

No statistically different changes in motor function, cognitive function, or perceived disability were observed between gamified DTT and STT, as revealed by the findings that the 84% CIs of the pre-post changes overlapped between the two training conditions. The lack of significant difference between training conditions may be attributed to the high inherent challenge in the STT. The STT in this study consisted of six real-life functional tasks that IWPD commonly experience difficulty in [47,48]. In addition, IWPD have been reported to demonstrate impaired automatic movement and to compensate by using explicit cognitive strategies when carrying out daily activities [49]. Thus, the STT in this study may already be cognitively demanding to IWPD. The presumed inherent challenge of STT is supported by the observed improvements in motor (frailty as measured by M-PPT) and cognitive (set shifting as measured by TMT B/A ratio) function induced by STT, as indicated by the findings that the 84% CIs for the changes in both measurements following STT were above zero. Thus, integrating cognitive training via the SMARTfit^®^ Trainer system to an already cognitively taxing physical training may not provide as much benefit as originally expected. Additionally, of note, both of the gamified DTT and STT protocols used the same set of functional tasks, which were specially designed to not only be task-specific, but to also allow variation in terms of three difficulty levels across five parameters (i.e., amplitude, endurance, balance, vision, and accuracy). This implies that the training outcomes of the gamified DTT were compared with those of a high standard of training intervention (in the form of the STT) rather than traditional, usual-care physical rehabilitation.

Even though the differences in the changes that were induced between the two training conditions did not reach statistical significance, the effect of gamified DTT in improving the MDS-UPDRS III scores appeared to be larger than that of STT (−3.88 versus 0.13 points for which a negative value indicates improvement in the MDS-UPDRS III score). The 84% CI for improvements in MDS-UPDRS III following gamified DTT did not overlap with zero, but such an improvement was not observed after STT. In addition, more participants achieved a MDC/MCID in their MDS-UPDRS III scores after gamified DTT than they did after STT (DTT_MDC_:STT_MDC_ = 4:2). This finding suggests that gamified DTT may provide an additional benefit in mitigating motor symptoms and modulating disease progression. The additional benefit of gamified DTT that was observed on motor function rather than on cognitive function is an interesting finding. Previous studies showed that compared to STT, DTT can promote automatic movement and reduce the interference of a secondary task on the primary motor task performance in IWPD [50,51]. Having the participant’s attention be devoted to a cognitive task during physical training may prohibit the use of compensatory cognitive strategies and may render the IWPD more reliant on the striatal motor pathway in movement execution, which may promote the restitution of the impaired striatal motor pathway [52,53]. These findings may reveal the potential importance of adding cognitive challenge into physical rehabilitation in IWPD who are in the mild to moderate disease stage.

In reference to MDC and/or MCID values, eight test scores of five participants (P1, P2, P3, P6, and P7) improved more than these clinically significant values after gamified DTT compared to the six test scores of three participants (P1, P5, and P7) after STT. Defining participants who improved more than the MDC and/or MCID values in at least one of the five outcome measures for either gamified DTT or STT as treatment responders for gamified DTT or STT, respectively, three categories of treatment responses can be discerned from these clinically relevant findings: (DS) two participants (P1 and P7) responded positively to both gamified DTT and STT, (D) three participants (P2, P3, and P6) seemed to respond well exclusively to gamified DTT, and (S) a favorable treatment response exclusive to STT seemed apparent for one participant (P5). As shown in Table A4 of Appendix B, the demographic and clinical parameters that are common to the participant (s) in the first two treatment response categories are (DS) non-faller and physically active and (D) less than two years of disease duration, faller, and high mobility confidence. As for the demographic and clinical characteristics of the single participant in the third response category (S) that can be cross-referenced with the other two response categories, they include a disease duration of less than two years, non-faller status, high mobility confidence, and physically inactive status.

### 4.1. Potential Predictive Characteristics for Differential Response to Gamified DTT versus STT

Is there any comparative advantage of gamified DTT relative to STT for particular subgroups within the PD population? Comparing the demographic and clinical characteristics of the three participants who responded most positively to only DTT (i.e., who belonged to the D treatment response category) with those of the one participant who responded the most positively to only STT (i.e., who belonged to the S treatment response category), the only clear distinction between these two categories is the former having faller status (D category) and the latter having non-faller status (S category). This comparative result may be indicative of faller status (defined as having had at least one fall event in a period of six months prior to study participation) as a potential viable classifier criterion for identifying a unique subgroup of IWPD who would benefit the most from gamified physical-cognitive rehabilitation relative to physical rehabilitation alone. Three participants (P2, P3, and P6) in the current cohort fit this fall history profile and indeed, they each improved in three outcome measures (two of which surpassed the MDC and/or MCID values for P2 and P6; one for P3) across at least two outcome domains (P2 and P3: motor, cognitive, and perceived disability; P6: motor and cognitive) after gamified DTT. In comparison, they responded less to STT, with two (P2 and P6) of them having each made improvements in only two outcome measures (all of which did not reach the MDC and/or MCID values) across at least one outcome domain (P2: cognitive; P6: motor and cognitive) and one (P3) of them having made no improvement in any of the five outcome measures. Importantly, the motor improvement for all these three participants (P2: 8 points; P3: 12 points; P6: 7 points) exceeded the MDC and MCID values (of 5 and 2.5 points, respectively) for MDS-UPDRS III—the gold standard established for assessing motor function in the PD population—upon the completion of gamified DTT, but not after STT. This finding indicating that IWPD with faller status respond more positively to therapeutic treatment involving gamified DTT may highlight the greater need for this subgroup of IWPD to receive training focused on improving their capacity for dual task performance. As indicated by the results of Heinzel et al.’s study showing a relationship between dual-tasking effects and fall risk for people with PD, higher deficiency in dual task performance in terms of walking 20 m in a straight line while checking boxes printed on paper (attached to a handheld clipboard) predicted a higher probability of future falls [54]. As a sidenote to the observations of the current study, the three participants in the D treatment response category received gamified DTT before STT, whereas the one participant in the S treatment response category underwent STT before gamified DTT (see Appendix B for details). Although the order of the two training conditions was randomly assigned to these four participants so as to achieve counterbalancing across all nine of the enrolled participants, the effect that the order of the training conditions might have on training effects could not be determined in the current study due to a statistical power limitation.

Among the participants who showed improvements in the outcome measures following either gamified DTT or STT, at least half of them tended to respond to one type of training condition rather than both training conditions. A study by Strouwen et al. (2019) showed that IWPD who had better cognitive function and slow gait velocity during dual tasking benefitted from DTT the most [55]. Together, these findings support that DTT individualized to each participant’s clinical characteristics is needed. For example, a person with PD who has mild cognitive impairment may benefit more from training requiring the simultaneous performance of a choice reaction task (of relatively low cognitive challenge) during multi-plane locomotion (of relatively complex physical challenge) than training involving performing a complicated arithmetic task during multi-plane locomotion, especially at an early phase of a training program. However, the sample size in this study is insufficient for subgroup analyses, and further research is needed to identify whether other factors or cut-off values can be used to identify those IWPD with certain clinical characteristics who may respond to gamified DTT or STT better.

It is also interesting to note that the only participant (P7) who presented with mild cognitive impairment and mild depression upon evaluation at the first visit was also assessed to have dementia, which was determined according to the PD-CRS score that was below 62 points at the first baseline [56]. Relative to the baseline PD-CRS score of 53 points (at T0), this participant subsequently reached and maintained a non-demented cognitive status by performing 40% better at the test session after the first training condition (with a score of 74 points at T1), which was STT, 34% better at the test session after the washout period (with a score of 71 points at T2), and finally 43% better at the test session after the second training condition (with a score of 76 points at T3), which was gamified DTT. There is paucity in the research literature of studies investigating training effects on the cognitive function of IWPD having MCI because cognitive dysfunction, as assessed by neurocognitive screening tests, is commonly used as an exclusion criterion for research participation involving the PD population, especially in fall intervention studies [57]. Hence, this unique case (P7) may be highly valuable in suggesting that it is beneficial for IWPD who have nonmotor symptoms affecting cognition and mood to receive some form of physical training, be it gamified physical-cognitive DTT or physical STT. This is because physical training with or without combining with cognitive training has the potential to not only reverse cognitive decline from the demented state to the non-demented state during initial training within the first 7.5 weeks (which is the length of STT period for P7), but that it also shows the potential of retaining some of that improvement in cognitive function for at least 11.5 weeks (which is the length of washout period for P7) after training, and to continue enhancing cognitive function during subsequent training in the last 7.5 weeks (which is the length of gamified DTT period for P7). With MCI being a risk factor for dementia, which is a major public health issue [58], training protocols that can be used as preventive measures or therapeutics to delay its onset, revert it back to normal cognition, or slow down its conversion to dementia will have important implications for alleviating individual, societal, and economic burden. It would also be interesting to investigate the potential training benefits of gamified DTT in treating mild depression and low mobility confidence (the other clinical characteristics unique to this participant, but not assessed as training outcome measures in the present study) in future studies.

### 4.2. Limitations

Some limitations of the present study include the small sample size with demographic and clinical heterogeneity (see Table 1 for details), so the findings for the current cohort cannot be generalized to other IWPD, and the poor statistical power, which restricts us from conducting sub-analyses to examine possible differences in treatment responses of disease subtypes. Due to the assessment of the outcome measures being conducted shortly after training with no follow-up assessments, the long-term retention of training benefits and detraining effects after a delayed period (e.g., six months, one year) are also unknown. As we did not use a direct measurement instrument for QoL, we could only infer the impact of each of the two training conditions on this outcome domain from changes in the participants’ self-assessment of their disability in relation to ADL, as measured by MDS-UPDRS II [24].

The amount of rest in between training bouts within each session was not controlled, so variable fatigue levels and training dosages in terms of the number of task repetitions and amount of time in active task practice might have influenced the results. However, allowing the participants to have control over their rest conditions, together with the choice of task order in both the gamified DTT and STT programs as well as options of cognitive tasks in the gamified DTT program, was intended as an autonomy support strategy for operationalizing the optimization of rehabilitation in this study [34]. Additionally, the physical therapists and investigators were not blinded to the study design or the assigned order of training interventions for the participants.

### 4.3. Potential Advantages and Implications in Traditional Clinical Setting and Telerehabilitation

Beyond considerations of feasibility, safety, and efficacy, the use of gamified DTT in neurorehabilitation has other potential advantages as a therapeutic intervention. In the neurorehabilitation of IWPD, interventions that employ technology such as exergaming and virtual reality equipment (e.g., Microsoft Kinect, Nintendo Wii-Fit) have either primarily targeted motor function alone or have included cognitive function as a secondary training outcome. In comparison, the gamified DTT used in the current study was specifically designed to influence functional outcomes in both the motor and cognitive domains. The use of the abovementioned technologies has also been constrained in their design adaptability to individual training needs [59,60]. The gamification of DTT as a combinatorial modality of delivering physical and cognitive training in an adaptive manner that can be tailored in real-time to suit individual needs rather than as a one-size-fits-all paradigm is a state-of-the-field, novel, and untapped potential of clinical significance.

The incorporation of game elements (e.g., score system, visual stimulation, auditory feedback, goals and targets, touch-response augmentation) in the dual task taxonomy used for neurorehabilitation has the potential to enhance engagement and motivation to ensure high-quality time spent on training through effort investment (with participants putting forth their best efforts to do well in the tasks) and program compliance (with high rates of adherence to training protocol and completion of the training regimen) [61,62], both of which can also contribute to greater data accuracy for the practice performance and training outcomes of the intervention.

Moreover, neurorehabilitation technology such as the SMARTfit^®^ Trainer system that can be pre-programmed with custom-designed programs containing task-specific activities for fast setup at any time during the course of a training session is valuable for facilitating more interaction time between the therapist and the patient. Within each training session, less time spent in setting up translates to more time available for attending to instructing, guarding, and observing patients for the therapists while increasing on-task training time (resulting in higher training dosage) for the patients. This up-scaling of time usage efficiency has the advantage of alleviating the physical burden that is placed on therapists of having to rush through the setting up of the task environment and/or having to forgo the enrichment of the task environment, for example, through gamification, in preference of a simple setup so as not to compromise therapist–patient interaction time or active training time for the patient.

In view of the contagion risks of implementing in-person PD neurorehabilitation during a period of heightened viral spread associated with close physical contact in a pandemic situation, the use of the SMARTfit^®^ Trainer system equipped with remote-access technology (e.g., Wi-Fi-enabled video monitors and audio communication devices) can offer physical therapists a valuable tool for leveraging technology to ensure the continuation of patient-centered therapy delivery in a pandemic-specific mitigation environment. For example, the physical therapist could be located in one room of a PT clinic equipped with audiovisual devices that are connected wirelessly to a PD patient who is located in a separate room that is also set up with a SMARTfit^®^ Trainer system equipped with network-connected communication capability within the same clinic, at another medical facility, or in the home environment of the patient. Specifically, with the use of the SMARTfit^®^ Trainer system for remote supervision-enabled training, patients can continue to receive individualized therapeutic treatment for their motor and cognitive symptoms with minimal disruption to their neurorehabilitation routine in times when reduced physical mobility, geographical relocation, or the occurrence of a viral pandemic situation prevents clinic visits.

Given the safety challenges of implementing physical therapy in a telerehabilitation-based setting for this patient population with postural instability as a hallmark of the disease [63], some modifications to the functional tasks used in the current study must be made to accommodate independence during patient training in the virtual presence of a physical therapist. For example, functional training permitting the use of stationary postures, such as a seated position or in-place standing, or involving dynamic postural changes close to the ground or a stable support platform, would be safer and more appropriate for implementation in a physically contactless or limited-contact therapeutic setting. For proof of concept, the findings of the current study can be used to inform the design of an adequately powered randomized controlled study to explore the feasibility, safety, and efficacy of utilizing gamified technology with time-efficient scalability and physical-distancing features to implement an adaptive, interactive, and effective DTT program for meeting person-specific functional training needs during PD telerehabilitation.

## 5. Conclusions

In a small cohort of PD patients, we found that gamified DTT implemented using SMARTfit training technology as a rehabilitation tool is feasible, safe, and appeared to show interesting directions to explore for improved efficacy compared to traditional therapeutic approaches. This article is the first to describe the training benefits of using a gamified training platform that is capable of facilitating visuo-tactile interaction during mental engagement for the implementation of physical-cognitive DTT in meeting the unique challenges of not only mitigating motor and cognitive symptoms simultaneously, but also perceived disability for IWPD. The use of neurorehabilitation technology in the form of the SMARTfit^®^ Trainer system by physical therapists in implementing a gamified DTT paradigm was demonstrated to be feasible for achieving a total of 24 training hours at a moderate exercise intensity, safely, with no incidence of adverse events.

## Figures and Tables

**Table 1:** Demographic, clinical, and baseline (T0) characteristics of enrolled participants ^a^.

Participant	Age (y)	Sex	Dx (y)	H&Y Stage	MoCA	HoF	ABC	GDS	LAPAQ	Perceived Disability	Motor Function		Cognitive Function	
										MDS-UPDRS II ^d^	MDS- UPDRS III ^d^	M-PPT ^e^	PD-CRS ^e^	TMT B/A ^d^
P1 ^b^	80	F	5.7	3	30	0	97.2	1	286	3	38	29	106	2.33
P2 ^c^	53	F	0.3	2	30	3	94.1	2	220	7	24	34	113	1.31
P3 ^c^	72	F	1.6	3	28	2	85.8	0	107	12	26	33	114	1.39
P4 ^c^	60	F	6.9	2	26	0	95.6	5	103	4	17	33	113	1.15
P5 ^b^	68	F	0.6	2	26	0	95.0	4	86	1	25	32	95	1.72
P6 ^c^	75	F	1.8	2	28	1	98.4	1	264	0	33	33	100	1.32
P7 ^b^	58	F	1.9	2	19 ^b^	0	76.3	19 ^c^	300	7	47	22	53	2.05
P8 ^c^	69	M	6.5	2	27	0	87.3	5	220	13	45	28	102	1.95
P9 ^d^	73	M	0.1	2	28	0	98.8	2	294	1	13	33	106	1.37

^a^ Dx = Time since diagnosis of disease; H&Y = Hoehn and Yahr Scale; MoCA = Montreal Cognitive Assessment; HoF = History of Falls Questionnaire (indicated here as the self-reported number of falls in the past six months before study participation); ABC = Activity-Specific Balance Confidence Scale; GDS = Geriatric Depression Scale (Long Form); LAPAQ = Longitudinal Aging Study Amsterdam Physical Activity Questionnaire (reported here as the total time per day in minutes). ^b^ Participant was assigned the single-task training condition followed by the dual-task training condition. ^c^ Participant was assigned the dual-task training condition followed by the single-task training condition. ^d^ Lower values indicate better scores for this outcome measure. ^e^ Higher values indicate better scores for this outcome measure.

## Data Availability

Available upon request.

## Appendix A

Details of the functional tasks and cognitive tasks used for implementing the gamified DTT and the STT are shown in Table A1, Table A2 and Table A3.

**Table A1:** Pairing of six functional tasks.

	Task A	Task B
Pair 1	Sit-to-stand	Multi-plane locomotion (obstacle course)
Pair 2	Gait	Reach and grasp
Pair 3	Floor-to-stand; stand-to-floor	Single limb standing

**Table A2:** List of 16 cognitive tasks from the SMARTfit^®^ Single system application.

Name of Game	Targeted Cognitive Processes									
	Selective Attention	Sustained Attention	Inhibitory Control	Cognitive Flexibility	Decision-Making	Visual Search	Visual-Perceptual Processing	Visuospatial Working Memory	Language Processing	Information Processing Speed
Category: Chase										
Chase the Color		1				2	1			1
Chase the Target		1	1	2		2	1			1
Category: Equations										
Addition, Subtraction	2	1				2				2
Squares, Division, Multiplication	2	2	2		1			1		2
Category: Memory										
Color, Numbers, Shapes		2				2		1	2	
Dice, Symbols		2				2		1		
Category: Seek										
Seek the Color	1		2			2				2
Seek the Letter	1			2		2			2	
Seek the Smiley	1			2		2				
Category: Track										
Track Left, Right, Both		2	1	2	2		1	2	2	
Track the Letter		1				2			1	2
Track the Color		1				2				2
Non-Categorized Games										
Lights Out		1					2			1
Pattern Recognition		2					1	1		
Rally		2					2			1
Spelling									1	

1 = Primary cognitive process. 2 = Secondary cognitive process.

**Table A3:** Difficulty levels of functional tasks.

Pair 1 Task A: Sit-to-Stand						
Level	Amplitude/Speed	Endurance	Balance		Vision	Accuracy
1	Perform task well enough so that the task is able to be completed in one attempt—18-inch height chair	Complete 10 repetitions	Both feet on the ground and allowed to use upper extremity support		No manipulation	Not using back of legs on the chair and standing up to full upright posture; no “toes up”
2	Perform task well enough so that the task is able to be completed in one attempt—16-inch height chair	Complete 20 repetitions	Both feet on the ground with goal of no upper extremity support		Look left/right	
3	Perform task well enough so that the task is able to be completed in one attempt—14-inch height chair	Complete 40+ repetitions	No upper extremity support with pelvis or feet on an unstable surface (i.e., DynaDisk cushion; Airex pad)		Eyes closed	Maintain end upright posture—no wobbling, no “toes up” end position
**Pair 1 Task B: Multi-plane locomotion (turns, stepping over, around, and between obstacles, steps, or stairs)**						
**Level**	**Amplitude/speed**	**Endurance**	**Balance**		**Vision**	**Accuracy**
1	Largest step at comfortable speed	15 min with any number of rest breaks	PT UE support	Stable surface but obstructed path with obstacles to walk around and over (short objects)	No manipulation	Not knocking over any obstacle
2	Maintain largest step length and increase speed	15 min with 1–2 rest breaks	Cane	Stable with obstacles to walk around and over (tall objects), and unstable surfaces (thick floor mat)	Look left/right	Predictable start and stops and 90° turns
3	Maintain largest step length and as fast as possible safely	15 min with no rest break	No Support	Stable with obstacles to walk around and over (tall objects), and unstable surfaces (step on Airex pads or river stones)	Scanning environment with head turns	Unpredictable start and stops and turns of 90° (2-steps), 180° (max of 4 steps), or 360° (max of 8 steps)
**Pair 2 Task A: Gait**						
**Level**	**Amplitude/speed**		**Endurance**	**Balance**	**Vision**	**Accuracy**
1	Largest step at comfortable speed		15 min with any number of rest breaks	Forward, side-ways, and backward walking with PT in front giving UE support	No manipulation	No accuracy manipulation
2	Maintain largest step length and increase speed		15 min with 1–2 rest breaks	Forward, side-ways, and backward walking with a cane	Look left/right	PT directed stop and start
3	Maintain largest step length and as fast as possible safely		15 min with no rest break	Forward, side-ways, and backward walking with no UE support	Scanning environment with head turns	Stop and start and regular turns (left, right, 180° or reversal from forward to back or side-stepping direction
**Pair 2 Task B: Reach and grasp**						
**Level**	**Amplitude/speed**		**Endurance**	**Balance**	**Vision**	**Accuracy**
1	Sitting—maximum excursion (DISTANCE) of the reach with variations in both HEIGHT: Shoulder height, up and down AND DIRECTION: forward, lateral, and across		10 reaches each arm	Blocked direction and blocked height	No manipulation	Grasp large object (e.g., water bottle) with palmer grasp
2	Standing—maximum excursion (DISTANCE) of the reach with variations in both HEIGHT: Shoulder height, up and down AND DIRECTION: forward, lateral, and across		20 reaches each arm	Blocked height variable direction	Look left/right	Grasp medium-sized object (e.g., highlighter) with tripod grasp
3	Standing: Step and reach Maximum excursion (DISTANCE) of the reach with variations in both HEIGHT: Shoulder height, up and down AND DIRECTION: forward, lateral, and across		40 reaches each arm	Variable height and direction	Look at object and then close eyes for reach and grasp	Grasp small object (e.g., paper clip) with pincer grasp
**Pair 3 Task A: Floor-to-stand; stand-to-floor**						
**Level**	**Amplitude/speed**		**Endurance**	**Balance**	**Vision**	**Accuracy**
1	With chair in front—down to knees and back up		5 repetitions	Using chair for upper extremity support	No manipulation	Best posture possible but OK if trunk is flexed; minimal sway or instability through the transition and in end positions
2	Down onto hands and knees and back up		10 repetitions	Not using chair	Look left/right	Maintenance of extension in trunk; minimal sway or instability through the transition and in end positions
3	All the way down into prone and back up		20 repetitions	Using least number of limbs possible (arms and legs)	Eyes closed	Maintenance of extension in trunk; minimal sway or instability through the transition and in end positions
**Pair 3 Task B: Single limb standing**						
**Level**	**Amplitude/speed**		**Endurance**	**Balance**	**Vision**	**Accuracy**
1	Lift one foot up slightly to hip/knee flexion 15°, and to hip abduction 10°		5 repetitions	Hand contact on an object as support	No manipulation	Maintain for 5 s per repetition
2	Lift one foot up to hip/knee flexion 30°, and to hip abduction 20°		10 repetitions	Hands on hip	Look left/right	Maintain for 10 s per repetition
3	Lift one foot up to hip/knee flexion 45°, and to hip abduction 30°		20 repetitions	With bilateral shoulder abduction/flexion 90°	Eyes closed	Maintain for 20 s per repetition

## Appendix B

Responders were participants who improved above the MDC and/or MCID values in at least one of the five outcome measures for either gamified DTT or STT, whereas non-responders were those whose improvements did not reach or exceed the MDC and/or MCID values in any of the five outcome measures for either gamified DTT or STT. Only four demographic and clinical characteristics (i.e., disease duration, fall history, balance confidence, and daily physical activity level) are shown here because they were found to be common amongst all the participants in either the DS treatment response category or the D treatment response category.

**Table A4:** Categorization of treatment responses (i.e, DS, D, and S), and common demographic and clinical characteristics of treatment responders within each category.

	Category of Treatment Response	Participant	Order of Training Conditions	Dx (y) ^a^	HoF ^b^	ABC ^c^	LAPAQ ^d^
Responders	DS	P1	STT-DTT	5.7	0 (NF)	97.2 (H)	286 (A)
		P7	STT-DTT	1.9	0 (NF)	76.3 (L)	300 (A)
	D	P2	DTT-STT	0.3	3 (F)	94.1 (H)	220 (A)
		P3	DTT-STT	1.6	2 (F)	85.8 (H)	107 (I)
		P6	DTT-STT	1.8	1 (F)	98.4 (H)	264 (A)
	S	P5	STT-DTT	0.6	0 (NF)	95.0 (H)	86 (I)
Non- Responders		P4	DTT-STT	6.9	0 (NF)	95.6 (H)	103 (I)
		P8	DTT-STT	6.5	0 (NF)	87.3 (H)	220 (A)

^a^ Dx = Time since diagnosis of disease. ^b^ HoF = History of Falls Questionnaire (indicated here as the self-reported number of falls in the past six months before study enrollment); F = faller status (due to having had at least one fall event in the six months prior to study participation); NF = non-faller status (due to having not fallen in the six months prior to study participation). ^c^ ABC = Activity-Specific Balance Confidence Scale; H = high mobility confidence (due to a score of at least 80.9 percentage points on the ABC scale; Powell and Myers, 1995); L = low mobility confidence (due to a score below 80.9 percentage points on the ABC scale; Powell and Myers, 1995). ^d^ LAPAQ = Longitudinal Aging Study Amsterdam Physical Activity Questionnaire (reported here as the total time of participation in [exercise and non-exercise] physical activities per day in minutes); A = physically active (due to having at least 150 min of physical activities per day; van Nimwegen et al., 2011); I = physically inactive (due to having less than 150 min of physical activities per day; van Nimwegen et al., 2011).

## References

[1] Parkinson’s Foundation Statistics. http://www.parkinson.org/Understanding-Parkinsons/Statistics.

[2] Poewe W., Seppi K., Tanner C.M., Halliday G.M., Brundin P., Volkmann J., Schrag A.E., Lang A.E. (2017). Parkinson disease. Nat. Rev. Dis. Primers.

[3] Postuma R.B., Aarsland D., Barone P., Burn D.J., Hawkes C.H., Oertel W., Ziemssen T. (2012). Identifying prodromal Parkinson’s disease: Pre-motor disorders in Parkinson’s disease. Mov. Disord..

[4] Bernal-Pacheco O., Limotai N., Go C.L., Fernandez H.H. (2012). Nonmotor manifestations in parkinson disease. Neurologist.

[5] Aarsland D., Larsen J.P., Tandberg E., Laake K. (2000). Predictors of nursing home placement in Parkinson’s disease: A population-based, prospective study. J. Am. Geriatr. Soc..

[6] Levy G., Tang M.X., Louis E.D., Côté L.J., Alfaro B., Mejia H., Stern Y., Marder K. (2002). The association of incident dementia with mortality in PD. Neurology.

[7] Aarsland D., Andersen K., Larsen J.P., Lolk A., Kragh-Sørensen P. (2003). Prevalence and characteristics of dementia in Parkinson disease: An 8-year prospective study. Arch. Neurol..

[8] Hely M.A., Reid W.G.J., Adena M.A., Halliday G.M., Morris J.G.L. (2008). The Sydney multicenter study of Parkinson’s disease: The inevitability of dementia at 20 years. Mov. Disord..

[9] Aarsland D., Bronnick K., Williams-Gray C., Weintraub D., Marder K., Kulisevsky J., Burn D., Barone P., Pagonabarraga J., Allcock L. (2010). Mild cognitive impairment in Parkinson disease: A multicenter pooled analysis. Neurology.

[10] Litvan I., Aarsland D., Adler C.H., Goldman J.G., Kulisevsky J., Mollenhauer B., Rodriguez-Oroz M.C., Tröster A.I., Weintraub D. (2011). MDS task force on mild cognitive impairment in Parkinson’s disease: Critical review of PD-MCI. Mov. Disord..

[11] Aarsland D., Creese B., Politis M., Chaudhuri K.R., Ffytche D.H., Weintraub D., Ballard C. (2017). Cognitive decline in Parkinson disease. Nat. Rev. Neurol..

[12] Williams-Gray C.H., Foltynie T., Brayne C.E.G., Robbins T.W., Barker R.A. (2007). Evolution of cognitive dysfunction in an incident Parkinson’s disease cohort. Brain.

[13] Wu T., Mark H. (2009). Dual task interference in Parkinson’s disease. US Neurol..

[14] Rizek P., Kumar N., Jog M.S. (2016). An update on the diagnosis and treatment of Parkinson disease. CMAJ.

[15] Emre M., Ford P.J., Bilgic B., Uc E.Y. (2014). Cognitive impairment and dementia in Parkinson’s disease: Practical issues and management. Mov. Disord..

[16] Sanchez-Luengos I., Balboa-Bandeira Y., Lucas-Jiménez O., Ojeda N., Peña J., Ibarretxe-Bilbao N. (2021). Effectiveness of cognitive rehabilitation in Parkinson’s disease: A systematic review and meta-analysis. J. Pers. Med..

[17] Fritz N.E., Cheek F.M., Nichols-Larsen D.S. (2015). Motor-cognitive dual-task training in persons with neurologic disorders: A systematic review. J. Neurol. Phys. Ther..

[18] Strouwen C., Molenaar E.A.L.M., Münks L., Keus S.H.J., Bloem B.R., Rochester L., Nieuwboer A. (2015). Dual tasking in Parkinson’s disease: Should we train hazardous behavior?. Expert. Rev. Neurother..

[19] De Freitas T.B., Leite P.H.W., Doná F., Pompeu J.E., Swarowsky A., Torriani-Pasin C. (2020). The effects of dual task gait and balance training in Parkinson’s disease: A systematic review. Physiother. Theory. Pract..

[20] Janssen J., Verschuren O., Renger W.J., Ermers J., Ketelaar M., van Ee R. (2017). Gamification in physical therapy: More than using games. Pediatr. Phys. Ther..

[21] Adcock M., Sonder F., Schättin A., Gennaro F., de Bruin E.D. (2020). A usability study of multicomponent video game-based training for older adults. Eur. Rev. Aging Phys. Act..

[22] Garcia-Agundez A., Folkerts A.-K., Konrad R., Caserman P., Tregel T., Goosses M., Göbel S., Kalbe E. (2019). Recent advances for Parkinson’s disease with exergames: A systematic review. J. Neuroeng. Rehabil..

[23] Li Z., Wang T., Liu H., Jiang Y., Wang Z., Zhuang J. (2020). Dual-task training on gait, motor symptoms, and balance in patients with Parkinson’s disease: A systematic review and meta-analysis. Clin. Rehabil..

[24] Rodriguez-Blazquez C., Rojo-Abuin J.M., Alvarez-Sanchez M., Arakaki T., Bergareche-Yarza A., Chade A., Garretto N., Gershanik O., Kurtis M.M., Martinez-Castrillo J.C. (2013). The MDS-UPDRS Part II (motor experiences of daily living) resulted useful for assessment of disability in Parkinson’s disease. Parkinsonism. Relat. Disord..

[25] Hughes A.J., Daniel S.E., Kilford L., Lees A.J. (1992). Accuracy of clinical diagnosis of idiopathic Parkinson’s disease: A clinico-pathological study of 100 cases. J. Neurol. Neurosurg. Psychiatry.

[26] Dalrymple-Alford J.C., MacAskill M.R., Nakas C.T., Livingston L., Graham C., Crucian G.P., Melzer T.R., Kirwan J., Keenan R., Wells S. (2010). The MoCA: Well-suited screen for cognitive impairment in Parkinson disease. Neurology.

[27] Yesavage J.A., Brink T.L., Rose T.L., Lum O., Huang V., Adey M., Leirer V.O. (1983). Development and validation of a geriatric depression screening scale: A preliminary report. J. Psychiat. Res..

[28] Powell L.E., Myers A.M. (1995). The activities-specific balance confidence (ABC) scale. J. Gerontol. A Biol. Sci. Med. Sci..

[29] van Nimwegen M., Speelman A.D., Hofman-van Rossum E.J.M., Overeem S., Deeg D.J.H., Borm G.F., van der Horst M.H.L., Bloem B.R., Munneke M. (2011). Physical inactivity in Parkinson’s disease. J. Neurol..

[30] King L.A., Horak F.B. (2009). Delaying mobility disability in people with Parkinson disease using a sensorimotor agility exercise program. Phys. Ther..

[31] Soke F., Guclu-Gunduz A., Kocer B., Fidan I., Keskinoglu P. (2021). Task-oriented circuit training combined with aerobic training improves motor performance and balance in people with Parkinson’s disease. Acta Neurol. Belg..

[32] van der Kolk N.M., King L.A. (2013). Effects of exercise on mobility in people with Parkinson’s disease. Mov. Disord..

[33] American Heart Association Target Heart Rates Chart. http://www.heart.org/en/healthy-living/fitness-basics/target-heart-rates.

[34] Wulf G., Lewthwaite R. (2016). Optimizing performance through intrinsic motivation and attention for learning: The OPTIMAL theory of motor learning. Psychon. Bull. Rev..

[35] Loftus G.R., Masson M.E. (1994). Using confidence intervals in within-subject designs. Psychon. Bull. Rev..

[36] Julious S.A. (2004). Using confidence intervals around individual means to assess statistical significance between two means. Pharmaceut. Statist..

[37] Goetz C.G., Tilley B.C., Shaftman S.R., Stebbins G.T., Fahn S., Martinez-Martin P., Poewe W., Sampaio C., Stern M.B., Dodel R. (2008). Movement Disorder Society-sponsored revision of the Unified Parkinson’s Disease Rating Scale (MDS-UPDRS): Scale presentation and clinimetric testing results. Mov. Disord..

[38] Schrag A., Sampaio C., Counsell N., Poewe W. (2006). Minimally clinically important change on the Unified Parkinson’s Disease Rating Scale. Mov. Disord..

[39] Shulman L.M., Gruber-Baldini A.L., Anderson K.E., Fishman P.S., Reich S.G., Weiner W.J. (2010). The clinically important difference on the unified Parkinson’s disease rating scale. Arch. Neurol..

[40] Brown M., Sinacore D.R., Binder E.F., Kohrt W.M. (2000). Physical and performance measures for the identification of mild to moderate frailty. J. Geron Med. Sci. A.

[41] King L.A., Wilhelm J., Chen Y., Blehm R., Nutt J., Chen Z., Serder A., Horak F.B. (2015). Effects of group, individual, and home exercise in persons with Parkinson disease: A randomized clinical trial. J. Neurol. Phys. Ther..

[42] Paschal K.A., Oswald A.R., Siegmund R.W., Siegmund S.E., Threlkeld A.J. (2006). Test-retest reliability of the physical performance test for persons with Parkinson disease. J. Geriatr. Phys. Ther..

[43] Pagonabarraga J., Kulisevsky J., Llebaria G., García_Sánchez C., Pascual-Sedano B., Gironell A. (2008). Parkinson’s disease-cognitive rating scale: A new cognitive scale for Parkinson’s disease. Mov. Disord..

[44] de Bobadilla R.F., Pagonbarraga J., Martínez-Horta S., Pascual-Sedano B., Campolongo A., Kulisevsky J. (2013). Parkinson’s disease-cognitive rating scale: Psychometrics for mild cognitive impairment. Mov. Disord..

[45] Mueller S.T., Piper B.J. (2014). The Psychology Experiment Building Language (PEBL) and PEBL Test Battery. J. Neurosci. Methods.

[46] Horváth K., Aschermann Z., Kovács M., Makkos A., Harmat M., Janszky J., Komoly S., Karádi K., Kovács N. (2017). Minimal clinically important differences for the experiences of daily living parts of movement disorder society-sponsored unified Parkinson’s disease rating scale. Mov. Disord..

[47] Brod M., Mendelsohn G.A., Roberts B. (1998). Patients’ experiences of Parkinson’s disease. J. Gerontol. B Psychol. Sci. Soc. Sci..

[48] Hariz G.M., Forsgren L. (2011). Activities of daily living and quality of life in persons with newly diagnosed Parkinson’s disease according to subtype of disease, and in comparison to healthy controls. Acta Neurol. Scand..

[49] Nonnekes J., Růžička E., Nieuwboer A., Hallett M., Fasano A., Bloem B.R. (2019). Compensation strategies for gait impairments in Parkinson disease: A review. JAMA Neurol..

[50] Jung S.H., Hasegawa N., Mancini M., King L.A., Carlson-Kuhta P., Smulders K., Peterson D.S., Barlow N., Harker G., Morris R. (2020). Effects of the agility boot camp with cognitive challenge (ABC-C) exercise program for Parkinson’s disease. NPJ Parkinson’s Dis..

[51] Yang Y.R., Cheng S.J., Lee Y.J., Liu Y.C., Wang R.Y. (2019). Cognitive and motor dual task gait training exerted specific training effects on dual task gait performance in individuals with Parkinson’s disease: A randomized controlled pilot study. PLoS ONE.

[52] Peterson D.S., King L.A., Cohen R.G., Horak F.B. (2016). Cognitive contributions to freezing of gait in Parkinson disease: Implications for physical rehabilitation. Phys. Ther..

[53] Takeuchi H., Magistro D., Kotozaki Y., Motoki K., Nejad K.K., Nouchi R., Jeong H., Sato C., Sessa S., Nagatomi R. (2020). Effects of simultaneously performed dual-task training with aerobic exercise and working memory training on cognitive functions and neural systems in the elderly. Neural. Plast..

[54] Heinzel S., Maechtel M., Hasmann S.E., Hobert M.A., Heger T., Berg D., Maetzler W. (2016). Motor dual-tasking deficits predict falls in Parkinson’s disease: A prospective study. Parkinsonism. Relat. Disord..

[55] Strouwen C., Molenaar E., Münks L., Broeder S., Ginis P., Bloem B.R., Nieuwboer A., Heremans E. (2019). Determinants of dual-task training effect seize in Parkinson disease: Who will benefit most?. J. Neurol. Phys. Ther..

[56] Marco S.D., Serrano M., Villena D., Granda D. (2017). Validation of the Parkinson’s Disease-Cognitive Rating Scale applying the Movement Disorder Society Task Force criteria for dementia associated with Parkinson’s disease. Mov. Disord. Clin. Pract..

[57] Domingos J.M., Godinho C., Dean J., Coelho M., Pinto A., Bloem B.R., Ferreira J.J. (2015). Cognitive impairment in fall-related studies in Parkinson’s disease. J. Parkinson’s Dis..

[58] Chapman D.P., Williams S.M., Strine T.W., Anda R.F., Moore M.J. (2006). Dementia and its implications for public health. Prev. Chronic. Dis..

[59] Barry G., Galna B., Rochester L. (2014). The role of exergaming in Parkinson’s disease. J. Neuroeng. Rehabil..

[60] Dockx K., Bekkers E.M.J., Van den Bergh V., Ginis P., Rochester L., Hausdorff J.M., Mirelman A., Nieuwboer A. (2016). Virtual reality for rehabilitation in Parkinson’s disease. Cochrane Database Syst. Rev..

[61] van Beek J.J.W., van Wegen E.E.H., Bohlhalter S., Vanbellingen T. (2019). Exergaming-based dexterity training in persons with Parkinson disease: A pilot feasibility study. J. Neurol. Phys. Ther..

[62] Lumsden J., Edwards E.A., Lawrence N.S., Coyle D., Munafò M.R. (2016). Gamification of cognitive assessment and cognitive training: A systematic review of applications and efficacy. JMIR Serious Games.

[63] Kim S.D., Allen N.E., Canning C.G., Fund V.S.C. (2013). Postural instability in patients with Parkinson’s disease. Epidemiology, pathophysiology and management. CNS Drugs.

